# The Time Knowledge Questionnaire for children

**DOI:** 10.1016/j.heliyon.2020.e03331

**Published:** 2020-02-06

**Authors:** F. Labrell, H. Câmara Costa, H. Perdry, G. Dellatolas

**Affiliations:** a Université Paris-Saclay, Université Paris-Sud, UVSQ, CESP, Inserm, 94807, Villejuif, France; b Grhapes (EA 7287), INSHEA (national Higher Institute for Training and Research on Special Needs Education), Suresnes, France

**Keywords:** Psychology, Time knowledge, School children, Assessment, Typically-developing children, Children with disabilities or disorders

## Abstract

Based on a definition of time knowledge as the correct representation and use of the various time units, a validated questionnaire, the Time Knowledge Questionnaire (TKQ) has been developed with norms for typically developing children aged 6–11 years. The TKQ is a relatively short (10–45 min) and innovative tool, comprising 25 questions broken down into 7 categories. The TKQ has good internal consistency. A total score and two summary scores are provided, assessing conventional time and estimative time respectively. A clinical application of the tool was shown to be of interest for children with disorders or disabilities.

## 1 Introduction

The question of time is not only relevant to the physical sphere but also to the psychological sphere. Albert Einstein^1^, answering a question from Henri Bergson about the links between psychology and physics during a meeting of the French Society of Philosophy in 1922, stated that time in human consciousness was not the time indicated by clocks. According to a current point of view, psychological time is our subjective relationship to physical time (Klein, 2009). However, our “subjective” time needs to be controlled by physical time to organize life in society (work, meals, leisure, etc.) (Fraisse, 1957), using conventional time unit systems, like clocks and calendars. To sum up, time processing covers various dimensions.

The development of time conceptions in typically-developing children has already been investigated in studies focusing, for instance, on time orientation (Friedman, 1983, 1984) or on the estimation of the duration of familiar daily activities (Friedman, 1990a). In addition, the development of the notion of time is often a cause for concern among children with learning disabilities and/or behavioral difficulties. Thus, it is important to provide valid evaluations and norms for conceptions of time among children of different ages, and to improve the conceptual and psychometric properties of existing tools.

This paper aims to provide a definition of the apprehension of time in school years that could be measured with a validated questionnaire covering various time components, as well as norms among typically developing children, for use among children with disabilities or disorders. After a review of the development of time conceptions during childhood and their origins, and a presentation of existing tools for the measurement of time conceptions among children, we describe the Time Knowledge Questionnaire (TKQ) which was recently elaborated on a two-dimensional basis.

## 2 Conceptual issues: the evolution of time conceptions

### 2.1 During the first months

The abstract concepts of time, number and space are related to concepts of *magnitude* (Walsh, 2003), which could be present at birth, before language acquisition or any extensive experience with time, number, and space (de Hevia et al., 2014). Infants aged 1 month seem to present a primitive sense of time, according to observations using a temporal conditioning of the pupillary reflex (Pouthas et al., 1993). Six and 10-month-olds were reported to be able to estimate the duration of an event learnt by habituation (VanMarle and Wynn, 2006; Brannon et al., 2007). More recently, temporal bisection tasks were used among 4-month-olds, suggesting an early ability to discriminate temporal intervals (Provasi et al., 2010). Overall, these paradigms suggest a very early ability to estimate short durations.

### 2.2 Time judgements without numbers and time units

Time judgments refer to the perception of short durations (seconds) in tasks that do not require verbal answers involving time units. Temporal reproduction tasks, for instance, improve during childhood (for a review see Droit Volet, 2016), with considerable inter-individual variability in time discrimination abilities in all age groups, associated with performance in other cognitive functions, such as attention (Hallez and Droit Volet, 2017), working memory and processing speed. Explicit time judgements become possible at about 3 years of age, when children receive temporal verbal instructions and can deliberately estimate the duration of a new event (Droit Volet, 2013). At 5 years old, when children are encouraged to count, they improve the accuracy of their judgments of duration (Clement and Droit Volet, 2006). However, before 5 years of age, children are still not aware of the passage of time, and time judgements are mostly context-dependent (Droit Volet, 2013).

### 2.3 The development of temporal concepts

According to a recent view (McCormack and Hoerl, 2017), very young children (from 18 months of age) think about locations in time according to familiar events within repeated sequences that occur at these locations (e.g the *brushing of teeth* is *after supper* and *before bedtime*), before they acquire an event-independent understanding of time. Four-year-olds are able to judge the relative order of two unrelated events 6 weeks appart (Friedman, 1991). At around 5 years of age, children become progressively able to assign a unique location in time for events occuring in the past, the present or the future, having acquired the concept of a linear and unidirectional time line (Tillman et al., 2017). From 5 years, explicit time knowledge emerges, based on everyday time judgments (Friedman, 1990b). Children can locate themselves from the present moment in relation to the past or the future: they acquire present-time awareness.

### 2.4 Mastering the conventional calendar and clock systems

From 5 years of age a slow developmental acquisition starts, allowing children to understand clocks and calendars, and to identify both repeated cycles (days, weeks, months) and unique times (e.g. the tenth birth-day). The verbal sequences of days and months are learnt in early primary school (Fraisse, 1957; Friedman, 1990b; Godart and Labelle, 1998), followed by the succession of years or seasons, between 7 and 8 years of age (Friedman, 1990a). Children first develop a list-based representation of the days and the months before being able to use an analogous spatial representation of time intervals between days and months (Friedman, 1983, 1984, 1986, 1990a). Most children manage to read both digital and analog clocks between 8 and 10 years (Burny et al., 2009). This requires explicit knowledge of the relationships between time units (*how many minutes in an hour?*) (Burny et al., 2011; Cohen et al., 2000; Friedman and Laycock, 1989), as well as numerical counting and mental calculation abilities.

Other aspects of time knowledge, such as the estimation of longer intervals, have been less widely investigated. For instance, the estimation of long intervals (longer than the seconds range) can involve: estimating the duration of an ongoing activity (e.g. in the case of an interview: *for how long have we been here together?*); questions about life-span, related to what is called diachronic thinking (e.g. *how long does it take to become a grandfather for a young adult?*); or time intervals concerning birthdays (e.g. *how long ago was your birthday?*). These evaluations of long durations should also be considered in investigations on time conception development, and probably depend on the same cognitive factors as other cognitive estimations of quantities, such as estimating *how many seeds there are in a watermelon* (Harel et al., 2007) (see below).

### 2.5 Potential sources of developmental changes in time processing

Three major potential sources of developmental changes in time processing, from outer to inner sources, can be identified. First, language and social experiences help children's understanding of time (Hudson, 2006). Through their own experience, children start to be able to represent, even implicitly at the beginning, the course of familiar sequences of events, for instance in their daily eating or washing routines (Nelson, 1996). With language acquisition, notions of time become explicit, particularly through shared discourse with adults, for instance when adults use the past tense. As Mc Cormack and Hoerl pointed out, “When parents and caregivers engage in talk about events at other times with children, they are essentially scaffolding children to begin to take different temporal perspectives on events” (2017, p.319).

Second, children's cognitive development leads to increasing reasoning about time dimensions (duration, sequences, etc.). According to Piaget (1969), when children develop a concept of quantifiable time, whatever the events, they start to calculate interval durations as well as the order of the sequences of an event. A recent study in typically developing children had shown that time knowledge depends on four numerical factors such academic knowledge of numbers and number facts, number line estimation (e.g., correspondence between a number and a distance), verbal working memory, and contextual estimation (e.g., the number 10 is few for “leaves on a tree”, but many for “children in a family”) (Labrell et al., 2016). In addition, a correct representation of time units (e.g. what is one minute?) also requires cognitive estimation, linked to everyday and contextualized activities (for example the time required to carry out an activity, e.g. *How long does it take to iron a shirt?,* see Harel et al., 2007). Cognitive estimation itself relies on other cognitive functions, especially executive functions, such as working memory, planning, inhibition, and self-correction. Different aspects of memory and executive functions, including selective attention, are major sources of age-related variance in time processing during childhood (Droit-Volet, 2013). Overall, the processing of long durations appears to be linked to attentional resources, and short-duration processing to short-term and working memory (Zélanti and Droit Volet, 2011). Four-year olds, for example, need working memory to understand sentences involving temporal prepositions, such as “before” and “after” (*before the girl took off her hat, she took off her coat*) (Blything et al., 2015).

Third, a neurobiological model of an internal clock has been suggested to explain time encoding (Matell and Meck, 2000). However, the specific location of this potential internal clock is still under debate, especially between the striato-frontal system and the striato-cerebello-frontal system (Droit Volet, 2013). The fronto-striatal region has been implicated among children with attention-deficit hyperactivity disorder (ADHD), who show significant timing deficits in temporal tasks (Toplak et al., 2006). The cerebellum has been implicated in time processing (Ivry and Keele, 1989), especially for short durations (Harrington et al., 2004). The estimation of the duration of daily activities has been shown to be less accurate among children with cerebellar tumors than among controls (Labrell et al., 2014; Labrell et al., 2017).

To sum up, the development of time processing has already been investigated for several time dimensions (estimation of durations, temporal concepts, orientation, mastering the clock and calendar, sense of time) and using several tasks, verbal or other, according to the children's age. However, while Piaget's first interest in time development was clearly organized around children's understanding of the relationships between time, speed and distance, more recent evaluations of time processing and understanding among typically developing children has often lacked a common theoretical background.

## 3 Time measurement tools for typically developing schoolchildren and for children with disability/disorders

Table 1 presents the few tools (including TKQ) available for time measurement during childhood.

**Table 1:** Some tools available for time measurement during childhood.

Tool, authors	Population, [age in years; months], number of participants	Scope	Time dimensions	Number of items	Type of assessment
Time Questionnaire for Children (TQC), Quartier (2009)	TDC, [6_13], 153	Time processing difficulties	Orientation, sequences, objective and subjective duration, planning	35	Questionnaire
Kit for assessing time processing activities (KaTid), Janeslätt et al. (2008)	TDC, [5_10], 144	Time processing activities	Time perception, time management, time orientation	61	Tabletop activities with supportive pictures
Test of Diachronic Thinking, Bouchere et al. (2007)	ASD, [7; 5_ 16], 23 TDC, [7; 3_15; 7], 23	Temporal cognition	Tendency, Transformation, Synthesis (Montangero, 1996)	3 categories of questions linked to each time dimensions	Questions about pictures representing temporal succession of states or events
Time Knowledge Questionnaire (TKQ), X (2016)	TDC, [6; 2_11; 1], 105	Conceptual knowledge in terms of time units	Orientation, sequences, time units, clock reading, life span, birthday, present time awareness	25	Questionnaire with pictured material

TDC: typically developing children; ASD: children with Autism Spectrum Disorder.

The Time Questionnaire for Children (TQC) was constructed in order to evaluate conceptions of time as well as to provide a useful screening for identifying time-processing difficulties (Quartier, 2009). Thirty-five items compose the TQC, assessing 5 dimensions: time orientation (e.g. *What month is it?*), sequences (e.g. *Can you tell me the order of the seasons?*), objective durations (e.g. *How long does it take when you brush your teeth?*), subjective durations (e.g. *Do you think 10 min is a long time to get to school?*), and planning (e.g. *How long will you be an adult?*). A factorial analysis evidenced 3 main factors (time orientation, sequences and planning) explaining only 25,4 % of the variance. The psychometric qualities of this questionnaire are not quite satisfactory according to the author himself.

Children with ADHD (Attention Deficit Hyperactivity Disorder) show time-processing deficits in terms of understanding a chronology (Barkley et al., 1997) or reproduction of short time intervals, involving working memory (Noreika et al., 2013; Smith et al., 2002). The KaTid (Kit for assessing Time Processing Ability) was constructed to measure three subcomponents of time processing activities identified as impaired among children with a disability like ADHD or autism spectrum disorder (ASD): experience of time (time perceptions), time orientation (location in time) and time management (allocating time to activities) (Wennberg et al., 2018; Janeslätt et al., 2013). The KaTid was validated on 144 typically developing children aged 5–10 years (Janeslätt et al., 2008). However, even if the KaTid is a reliable tool, it contains a lot of items (N = 61) making the screening arduous, especially for young children.

Temporal cognition in children with ASD has already been investigated in terms of *diachronic thinking* (Montangero, 1996). This ability, not widely studied so far, enables changes occuring across time to be represented and understood (e.g. in a living creature, human or otherwise). As for most children with ASD the passage of time is not linked to ongoing activities, it seemed relevant to investigate diachronic thought in this population (Boucher et al., 2007). Three dimensions of diachronic thinking were evaluated in the cited study, such as Tendency (evoking past or future stages of a current situation), Transformation (understanding that qualitative changes over time do not alter one's identity) and Synthesis (understanding that temporal successions of different states/events are compressed into a whole unit, as is the case with the succession ofseveral modes of transport when travelling far away on holiday) (Maurice-Naville and Montangero, 1992; Montangero, 1996; Pons and Montangero, 1999). However, even if this evaluation provides an accessible assessment of a little-studied time dimension, diachronic thought, the small size of the sample does not enable any norms to be proposed.

To sum up, the currently available tools for time processing during childhood do not provide a valid evaluation because of the small numbers of participants, the length of the tool, or the poor psychometric properties of the scales proposed. In addition, the available tools do not enable time scores to be interpreted in relation to norms. These are the reasons why we developed the Time Knowledge Questionnaire.

## 4 The Time Knowledge Questionnaire (TKQ)

Time knowledge (TK) has been defined “as the correct representation and use of the various time units (e.g., seconds, minutes, hours, days, weeks, months, seasons, years” (Labrell et al., 2016, p.2). Indeed, time knowledge, according to Friedman (1990b), refers to a primitive sense of time as well as to the conventional system of time units which help children to deal with life in society (school activities, meals, leisure, etc.). As far as we know, no tool for the measurement of TK among school children has been developed yet, even if several time dimensions (time orientation, telling the time on a clock, diachronic thinking, etc.) have already been separately investigated (see Table 1).

**Figure 1:**
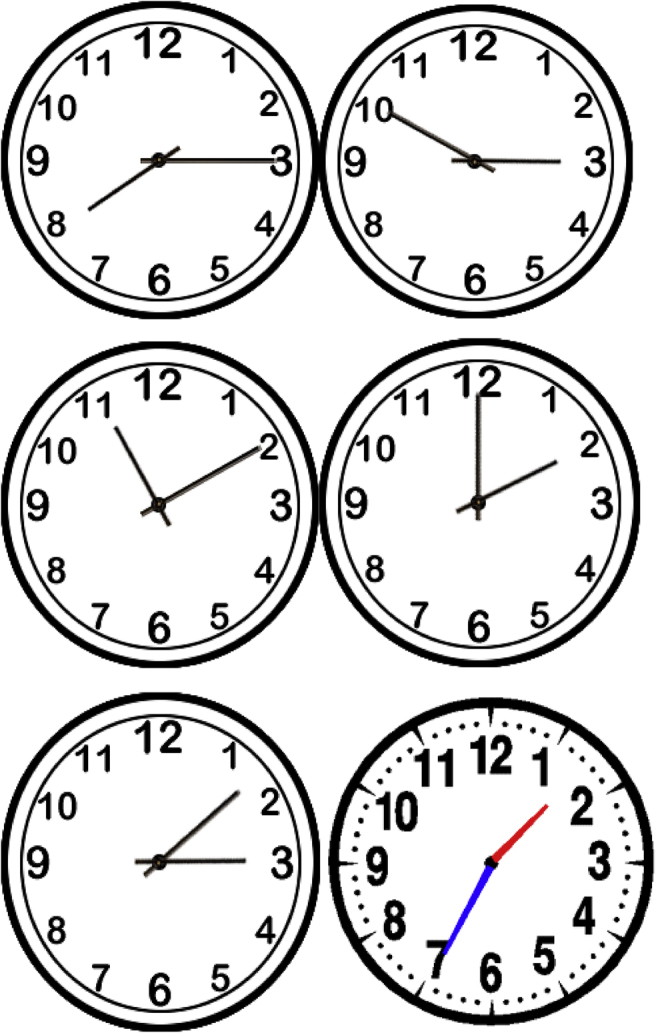
Material for the Telling the time on a clock (CL) subtest. (1) *Can you show me 2 o'clock?*; (2) *Can you show me 10 to 3?*; (3) *Can you show me a quarter past 8?*; (4) *Can you show me 10 past 11?* (5) *Look at this clock (with the red hand*). How many minutes is it to 2 o'clock?

**Figure 2:**
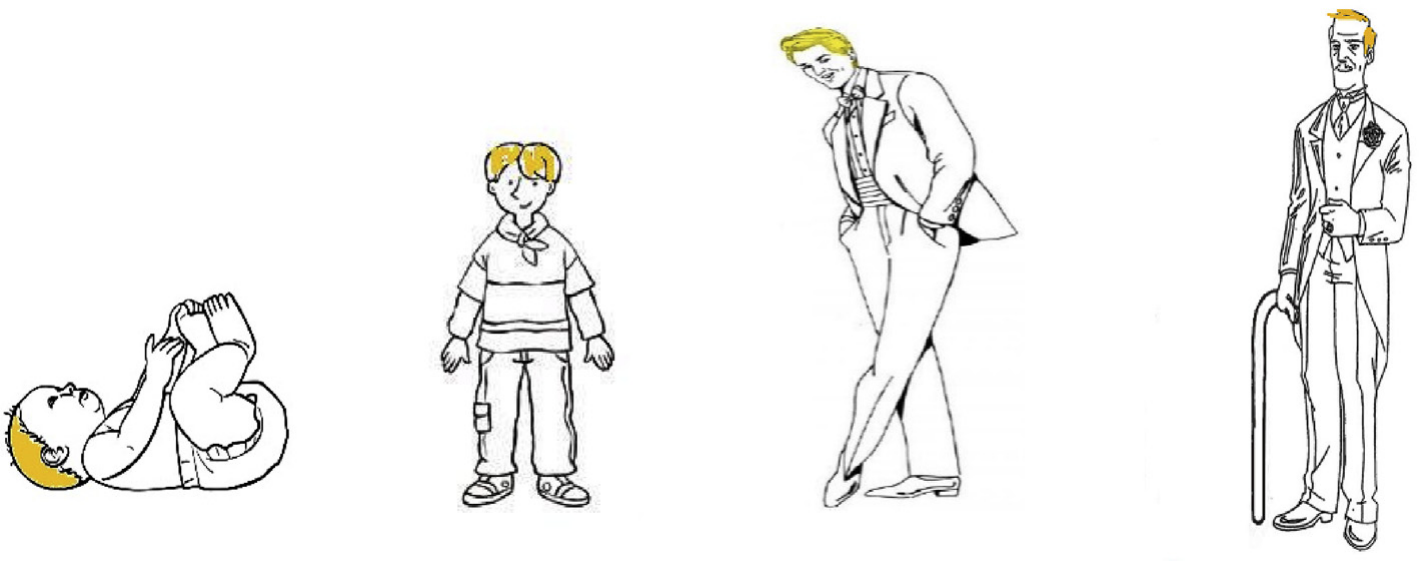
Material for the Life Span (LS) subtest. *How long does it take: (1) for a baby (here) to become a child (here)? (2) for a child (here) to become a young man (here)? (3) for a young man to become an old man?*

The TKQ comprises seven subtests. The first four subtests investigate conventional time knowledge: orientation (OR), sequences (SEQ), time units (TU), and telling the time on a clock (CL), comprising 5, 3, 4, and 5 questions respectively. The sum of these four subscores provides a conventional time score. The fifth subtest (3 questions), life span (LS), involves diachronic thinking. The sixth subtest (four questions) involves birthdays (BIR). Last, the seventh subtest (one question), interview duration (ID), investigates the estimation of the duration of an ongoing activity. Telling the time on a clock (CL) and life span (LS) both require pictures to be commented on by the child (Figure 1 and Figure 2). As in other assessments, the TKQ requires verbal answers. However, all questions were short and clear (see AppendixA), and children's answers were encouraged by a pictured material (clocks and characters, see Figure 1 and Figure 2 respectively).

## 5 Method

### 5.1 Participants

#### 5.1.1 Typically-developing sample

The participants were 105 school children from Grade 1 to Grade 5, 57 girls (54%) and 48 boys (46%), aged 6.2–11.1 years. Each school grade corresponds to a different age range (see Table 2). Children were recruited in their schools, with informed consent to participate in the study obtained from both parents. The participants were recruited via state schools and teachers asked children with no special educational needs or behavioral or neurological difficulties to join the sample. This sample aimed at representing the French inhabitants living in a relatively large city (200,000 inhabitants) near Paris. They came 50% from the middle social classes, 28% from the upper social classes and 22% from the lower social classes. It is important to note, however, that this sample is not nationally representative, although it is comparable to the typical population found in urban areas in Metropolitan France.

**Table 2:** Participants’ school grade, gender and age.

School grade	Age group (years)	*N*	Girls [n (%)]	Age in years [mean (SD), range]
1	6	17	8 (47)	6.41 (0.14), 6.2–6.7
2	7	19	12 (63)	7.52 (0.13), 7.2–7.7
3	8	22	11 (50)	8.66 (0.25), 8.1–8.95
4	9	28	13 (46)	9.44 (0.32), 9.0–9.97
5	10	19	13 (68)	10.58 (0.29), 10.1–11.1
Total		105	57 (54)	8.65 (0.40), 6.2–11.1

These participants have been tested with the Zareki-R, a battery for the evaluation of number processing and mental calculation (Labrell et al., 2016).

#### 5.1.2 Clinical sample

For the purpose of examining the applicability of the TKQ to a clinical sample, we recruited subjects who had been treated for a malignant cerebellar tumor in the Pediatric Oncology Department in the Gustave Roussy Hospital in Villejuif, France. Indeed, the role of the cerebellum in time processing has been generally acknowledged (Ivry and Keele, 1989).

To be included in this sample children had to present no relapse history, to be out of treatment and to be at least 6 years old at the time of assessment. The resulting sample was composed of 38 participants [14 girls (37%)], aged 6.1–20.4 years at the time of assessment (age at diagnosis = 0.1–18 years), whose treatement for a medulloblastoma (*n* = 34) or an ependymoma (*n* = 4) included surgery, radiotherapy and/or chemotherapy (time lapse since diagnosis = 0.1–14.9 years). All children were living in urban areas in Metropolitan France and French overseas territories, and came from middle- and upper-middle-class backgrounds (cf. Labrell et al., 2017, for further details on the composition of the clinical sample).

### 5.2 Procedure

The study was approved by the ethics committees at both participating institutions, the Gustave Roussy Hospital and the university of Reims (France).

Each child was separately interviewed by a trained experimenter in a quiet room at her/his school. Sessions lasted approximatively 15 min for typically-developing children. The children had no time limit for answering questions using the pictures for CL (see Figure 1) and LS (see Figure 2). However, the experimenter recorded the time at the beginning of the interview, and also at the end, in order to compute the error margins for the final interview duration answer in the ID subtest. The child's answers were fully transcribed by the experimenter.

### 5.3 Coding conventional time knowledge (score): OR, SEQ, TU, CL (see AppendixA)

Each answer was coded as correct (1 point) or incorrect (0 point), for a total conventional time score ranging from 0 to 17.

The questions about telling the time (CL) on a clock used pictures of five analogue clocks with five different positions of the minute and hour hands (see Figure 1).

### 5.4 Coding LS

This subtest uses four pictures (a baby, a child, a young man, and an old man) illustrating the biological process of ageing (see Figure 2). A fair-haired man, depicted at four different stages in development from infancy to old age, was chosen. The child was asked three questions about the time required to go from one age to the next (i.e. from baby to child, from child to young man, and from young man to old man). Each answer was coded 0, 1, or 2 points.

The coding rule was based on the answers of 20 adults, which were as follows: from baby to child, median = 6 years, range = 4 to 10; from child to young man, median = 22 years, range = 15 to 30; from young man to old man, median = 35 years, range = 20 to 55. Two points were given if the child gave an answer in the adult range. One point was given if the answer was borderline (i.e. not in the adult range but not clearly impossible), that is, within the following intervals: baby–child, 2 to 3.99 and 10.01–15 years; child–young man, 5 to 14.99 and 30.01–40 years; young man–old man, 55.01–90 years. No points were given for answers outside these ranges or for "don't know" answers (total LS score from 0 to 6).

### 5.5 Coding BIR

Questions 1 to 3 (*How old are you*?, *How old were you last year*?, *How old will you be next year*?) were not taken into account in the calculation of the score because the answers were invariably “correct” (i.e. the absolute difference between the given age and the exact age was always less than 1 year). The other three questions regarding birthdays were “*When is your birthday*?”, “*How long ago was your birthday*?”, and “*How long is it to your next birthday*?” For these three questions, the correct answer was available (for each child, the date of birth and date of interview were recorded), which made it possible to use a coding for error. Zero point were given if the child did not know the date of his or her birthday. Otherwise, for the last two questions (i.e. time intervals to the previous and next birthdays), 0 point were given for “*don't know*” and answers exceeding 12 months, and 1, 2, and 3 points were given for errors (i.e. absolute difference between the answer and the correct interval) greater than 3 months, between 3 months and 1 month, and less than 1 month, respectively (i.e. total BIR score ranging from 0 to 6).

### 5.6 Coding ID

At the end of the interview, the examiner asked the child, “*For how long have we been here together?*” The median duration of the interview was 15 min (inter-quartile range = 14–21 min, range = 5–42). Zero point were given for “*don't know*” answers. For all other answers, 0 to 10 points were given according to the absolute difference between the log of the real duration of the interview and the log of the answer (in minutes).

AppendixB presents the scoring grid for this subtest, which allows the child's answer to be scored according to the correct duration of the interview. As an example, if the real duration of the interview was 10 min and if the child answered that the interview had lasted between 8 to 10 min, then the answer was given a score of 10. Conversely, for the same interview duration of 10 min, if the child answered "*don't know"* or that the interview had lasted 1 min or more than 1 h, then the answer was coded 0.

## 6 Statistical analysis

We created two summary scores: the first four subtests (Orientation, Sequences, Time Units and Telling the time on a clock) were summed to create Summary Score 1 (Conventional time summary score); the remaining three subtests (Life-Span, Birthdays and Interview duration) were added to form Summary Score 2 (Estimative time summary score). Statistical analyses focused on the internal consistency of these scores, and their correlations after adjusting for age. Norms by grade (mean, SD, range, quartiles) are presented for all subtests, summary scores and the total score. Data from a clinical sample (children treated for a cerebellar tumor) was compared with these norms.

## 7 Results

### 7.1 Descriptive statistics of the TKQ

Table 3 presents the means, standard deviations, ranges and quartile distribution of all items in the TKQ subtest, as the well as the Summary and Total scores for each grade and for the whole sample. There were generally no floor effects, with the exception of the Birthday subtest, and minimum-to-moderate ceiling effects on the conventional time subtests (Orientation, Sequences, Time Units and Telling the time).

**Table 3:** Subtest descriptive statistics in the Time Knowledge Questionnaire.

	Grade 1	Grade 2	Grade 3	Grade 4	Grade 5	Total
**OR**						
mean (sd)	3.6 (1.2)	4.7 (0.6)	4.9 (0.3)	4.8 (0.4)	5.0 (0.0)	4.6 (0.8)
Range	1–5	3–5	4–5	4–5	5–5	1–5
Q1, Q2, Q3	3, 4, 5	4, 5, 5	5, 5, 5	5, 5, 5	5, 5, 5	5, 5, 5
**SEQ**						
mean (sd)	0.9 (0.8)	2.2 (1.0)	2.4 (0.8)	2.5 (0.6)	2.9 (0.3)	2.2 (0.9)
Range	0–3	0–3	1–3	1–3	2–3	0–3
Q1, Q2, Q3	0, 1, 1	2, 2, 3	2, 3, 3	2, 3, 3	3, 3, 3	2, 3, 3
**TU**						
mean (sd)	1.7 (0.8)	3.1 (0.8)	3.7 (0.7)	3.8 (0.5)	3.9 (0.2)	3.3 (1.0)
Range	0–3	2–4	1–4	2–4	3–4	0–4
Q1, Q2, Q3	1, 2, 2	2, 3, 4	4, 4, 4	4, 4, 4	4, 4, 4	3, 4, 4
**CL**						
mean (sd)	2.1 (1.6)	3.7 (1.0)	3.7 (0.9)	4.3 (0.6)	4.9 (0.3)	3.8 (1.3)
Range	0–4	1–5	1–5	3–5	4–5	0–5
Q1, Q2, Q3	1, 2, 4	4, 4, 4	3, 4, 4	4, 4, 5	5, 5, 5	4, 4, 5
**Summary score 1 (S1)**						
mean (sd)	8.3 (2.5)	13.7 (2.4)	14.7 (1.9)	15.4 (1.2)	16.7 (0.6)	14.0 (3.2)
Range	4–12	9–17	10–17	12–17	15–17	4–17
Q1, Q2, Q3	6, 9, 10	12, 14, 16	14, 16, 16	15, 16, 16	17, 17, 17	12, 15, 16
**LS**						
mean (sd)	2.1 (2.0)	3.7 (2.2)	4.8 (1.5)	4.6 (1.4)	5.2 (0.8)	4.2 (1.9)
Range	0–6	0–6	1–6	0–6	3–6	0–6
Q1, Q2, Q3	0, 2, 3	2, 4, 6	5, 5, 6	4, 5, 6	5, 5, 6	3, 5, 6
**BIR**						
mean (sd)	1.6 (2.0)	3.8 (2.0)	4.7 (1.5)	5.2 (1.1)	5.5 (0.7)	4.3 (2.0)
Range	0–5	0–6	2–6	2–6	4–6	0–6
Q1, Q2, Q3	0, 0, 3	2, 4, 6	3, 5, 6	5, 6, 6	5, 6, 6	3, 5, 6
**ID**						
mean (sd)	4.0 (3.6)	5.7 (3.6)	8.2 (1.5)	7.1 (2.7)	8.9 (1.2)	6.9 (3.1)
Range	0–9	0–10	5–10	0–10	7–10	0–10
Q1, Q2, Q3	0, 4, 8	2, 7, 9	7, 8, 9	6, 8, 9	8, 9, 10	6, 8, 9
**Summary score 2 (S2)**						
mean (sd)	7.6 (5.5)	13.2 (5.6)	17.6 (3.0)	16.9 (3.1)	19.5 (2.0)	15.4 (5.5)
Range	0–16	1–22	11–22	10–22	14–22	0–22
Q1, Q2, Q3	4, 8, 12	11, 13, 17	16, 18, 20	15, 17, 19	18, 20, 21	13, 17, 19
**TKQ (S1+S2)**						
mean (sd)	15.9 (6.0)	26.9 (7.0)	32.3 (4.1)	32.3 (3.3)	36.3 (2.1)	29.4 (8.0)
Range	4–24	11–37	22–38	25–38	31–39	4–39
Q1, Q2, Q3	13, 15, 21	26, 28, 31	29, 33, 35	31, 33, 33	35, 37, 38	26, 32, 35

Legend: Q1 = 25 %, Q2 = 50%, Q3 = 75 %.

Summary scores 1 and 2 both increased with participants' age. The Summary scores were significantly correlated one to another (Pearson *r* = 0.66, p < 10⁻^1^³), and correlated significantly with the participants’ age (*r* = 0.75, p < 10⁻^1^⁵ for Summary score 1 and *r* = 0.65, p < 10⁻^1^³ for Summary score 2).

**Figure 3:**
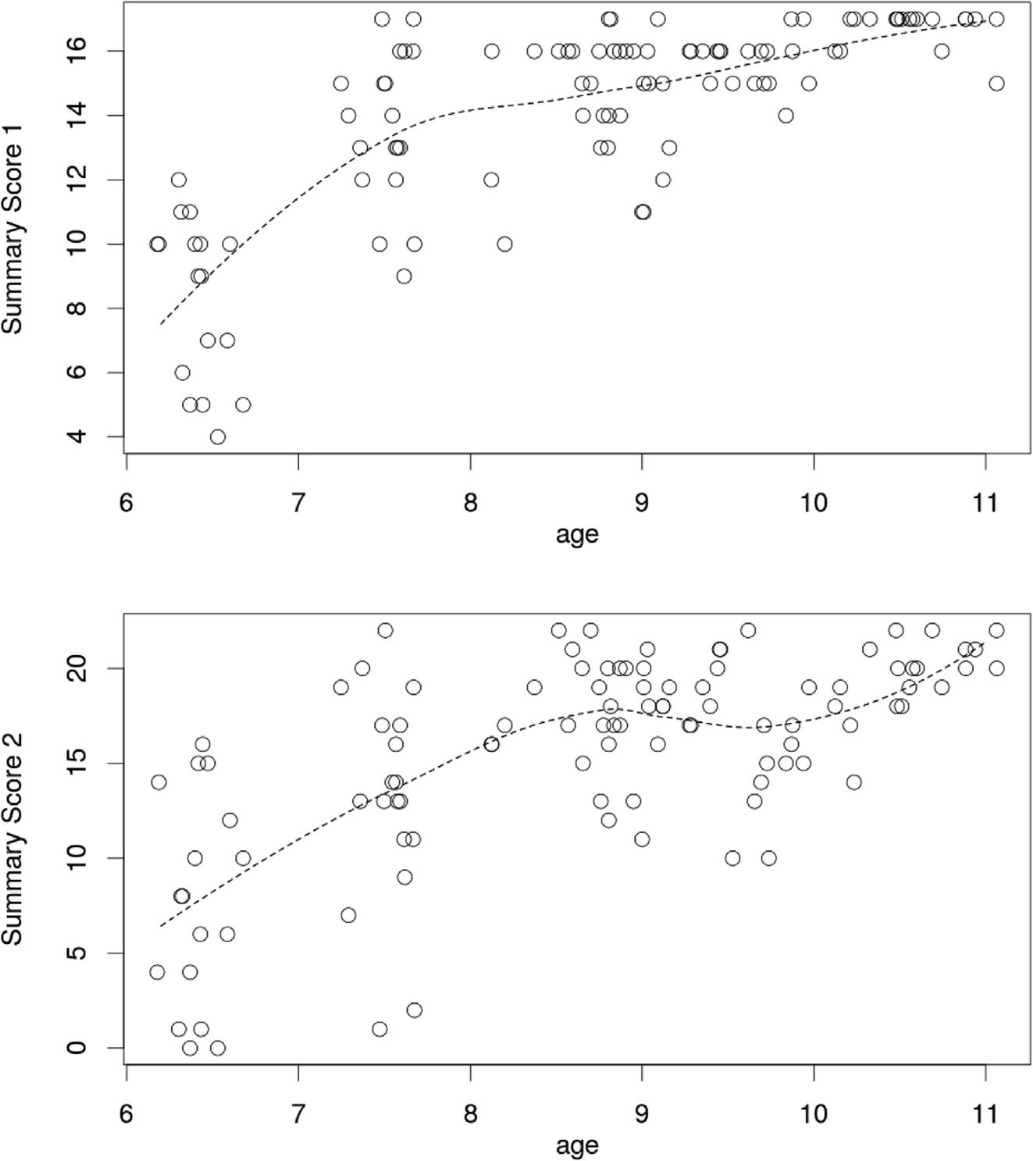
Distribution of Summary scores 1 and 2 according to the participant's age. The dotted line is the score predicted at a given age by loess regression.

A large part of the correlation between S1 and S2 was attributable to the fact they shared age as a common factor. To get an estimate of the correlation that was not attributable to this factor, we performed local loess regressions on S1 and S2 on age, using the R function "loess" (Cleveland et al., 1992; R Core Team, 2018). The correlation between the residues of these regressions was r = 0.25 only (see Figure 3), showing that S1 and S2 measure two different aspects of time knowledge.

### 7.2 Internal consistency

Internal consistency reliability coefficients for the two summary scores on the TKQ were in the acceptable range, with the following Cronbach alpha coefficients: .76 for Summary Score 1, .70 for Summary Score 2 and .76 for the Total score.

### 7.3 Construct validity and application to a clinical sample

**Figure 4:**
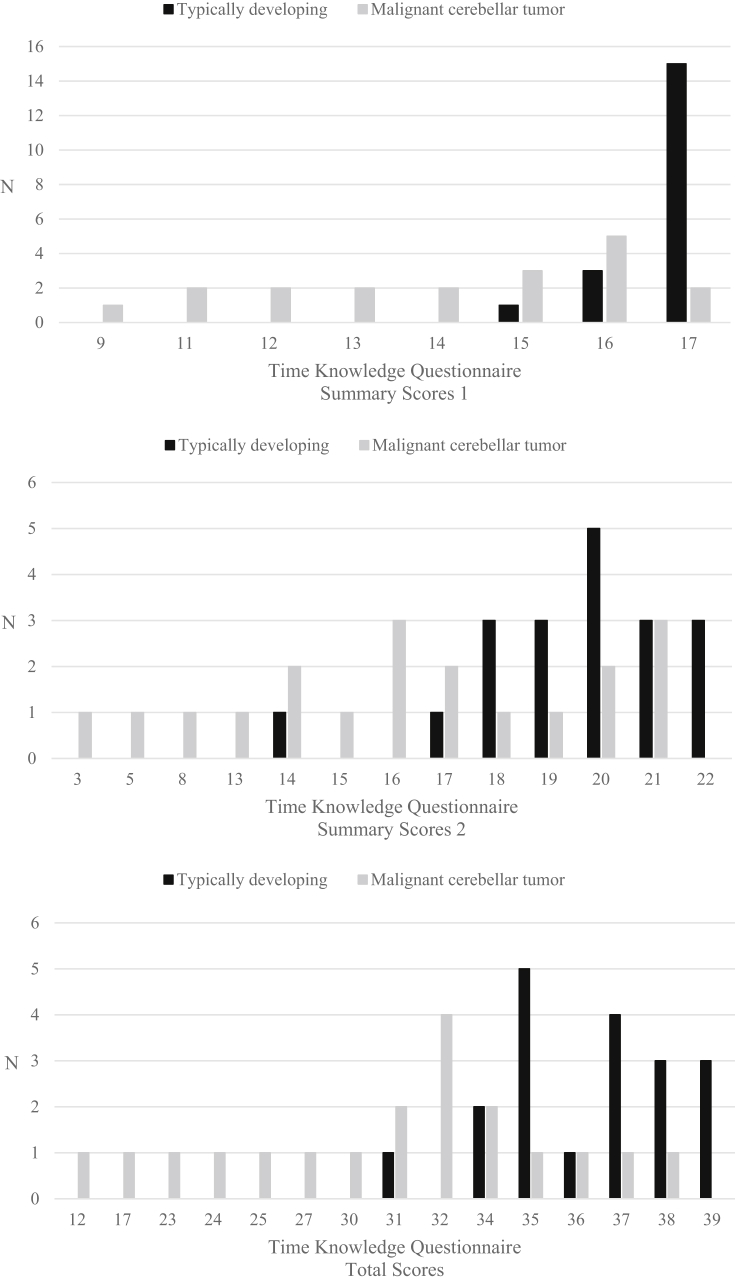
Distribution of the Time Knowledge Questionnaire Summary and Total scores: typically developing sample and malignant cerebellar tumor sample.

Figure 4 presents the distribution of the Summary and the Total scores on the TKQ in a sample of typically-developing children and in a sample of children who had had a malignant cerebellar tumor.

There was a significant number of children from the malignant cerebellar tumor sample whose performance was below the lowest score observed in the typically developing sample: Summary Score 1: n = 12 (63%)*vs*. n = 1 (5%) [lowest score = 15]; Summary Score 2: n = - (32%) *vs*. n = 1 (5%) [lowest score = 14]; Total score: n = 9 (47%) *vs*. n = 1 (5%) [lowest score = 31].

Compared to the malignant cerebellar tumor sample, children from the typically-developing sample exhibited better scores on Summary score 1 [M(SD) = 16.74 (0.56) *vs*. 14.11 (2.28), *t* = 4.88, *p*=<.0001), Summary Score 2 [M(SD) = 19.53 (1.98) *vs*. 15.47 (5.21), *t* = 3.17, *p* = .0043) and the Total score on the TKQ [M(SD) = 36.26 (2.10) *vs*. 29.58 (6.8), *t* = 4.09, *p*=<.0005), although participants in the typically-developing sample were significantly younger than those in the malignant cerebellar tumor sample [M(SD) = 10.58 (0.29) *vs*. 12.34 (1.43), *t* = -5.28, *p*=<.0001).

For the malignant cerebellar tumor sample, there were no significant associations of the TKQ Summary and Total scores with the Wechsler scale indices, namely the Full Scale Intellectual Quotient (IQ), Verbal IQ, Performance IQ and Processing Speed index (Pearson's *r* range = .05 to .36, *p* > 0.05 in all cases). The Working Memory Index was moderately correlated with TKQ scores (*r* = .33, *p* < .05). Results show altogether that time difficulties of children with malignant cerebellar tumors could not be completely explained by lower IQ (Labrell et al., 2017).

## 8 Discussion

In order to provide a validated questionnaire measuring time knowledge in the school years, the TKQ was designed for children aged from 6 to 11 years. Statistical analyses enabled an overall total score to be generated and norms to be provided from typically-developing children in the measurement of time knowledge. This means that school children's time knowledge can now be assessed with an innovative tool comprising amusing and varied questions, with a short administration duration (from 10 min for the older children to 45 for the youngest). In addition, our analyses also provided two summary scores with satisfactory internal consistency: one score for conventional time, based on true/false answers (the answer was correct or not, without intermediate possibilities), S1, containing four subtests (Orientation, Sequences, Time Units and Telling the time on a clock) and another score, S2, comprising three subtests (Life Span, Birthdays and Interview duration) for time estimation (the answer is more or less correct). S1 has already been measured in terms of sequences and orientation in the TQC (Quartier, 2009) but the poor psychometric properties of this questionnaire did not enable norms to be provided. In the current version, S1 is a relevant subscore based on numerical abilities and knowledge acquired at school, in relation to language, automatic sequences and relationships between time units (e.g. *how many seconds in a minute?*) (Labrell et al., 2016). Indeed, this kind of time dimension is in the curriculum as early as primary school, either for the knowledge of words expressing time (*days of the week, months of the year, etc…*) or time computation (*how many minutes are there until the 9 pm film if it is 5.45 pm?*). On the other hand, S2 which entails time estimations, has never been used, as far as we know, in a time questionnaire for children, whereas its relevance has already been shown in terms of “sense of time” (Harel et al., 2007).

Therefore, time perception during childhood could also be linked to time estimation, which is a kind of sense of time that has not been learnt and that has been shown to be linked to achievement in mathematics at school (Halberda et al., 2008). The S2 score was only moderately correlated to the S1 score (r = 0.25) after controlling for age, suggesting that it measures, to some extent, skills that are not captured by S1; thus, a total score could underestimate specific skills such as cognitive estimation and numerical abilities. Moreover, the lower performances observed in the TKQ by children with malignant cerebellar tumors are congruent with the role of the cerebellum in time processing (Ivry and Keele, 1989). Furthermore, the absence of strong correlations between the TKQ scores and the Wechsler scale indices (except a moderate correlation with the WMI) is in favor of the specificity of the timing processing difficulties in children after cerebellar damage.

In other words, the two subscores of the TKQ allow clinical applications. It would be for instance interesting to compare conventional and estimative time in case of a low total score. Indeed, if the conventional score is low, it could be relevant to schedule orthophonic interventions for numeracy, calculation or language in order to enhance the child's time knowledge. More generally, the TKQ could be useful for children with different disabilities, such as children with ASD whose sense of passage of time seems poorly related to ongoing activities (Boucher et al., 2007). Would they have a lower estimative score (S2) than typically-developing children? Furthermore, would children with ADHD have a lower conventional time score (S1) than typically developing children, while they have been shown to have difficulties in understanding chronology (Barkley et al., 1997)?

Lastly, our study also presents limitations that could be improved upon. First of all, the TKQ questions involve verbal answers and, thus, this instrument might not be adapted for children presenting significant verbal comprehension deficits. Further research on typically developing children from contrasted educational backgrounds, as well as on clinical samples, might answer this question. Furthermore, the results for children who have survived malignant cerebellar tumors have shown a possible implication of working memory in time knowledge processing, as is the case in number processing (Labrell et al., 2016). Working memory could be the executive function children need to answer the different subtests in the TKQ correctly (i.e. orientation, present-time awareness, telling the time on a clock, birthdays, life span and the duration of the interview). Future investigations are also needed in order to examine the associations between time knowledge and other cognitive components, such as language skills and memory, during development.

To summarize, time knowledge defined as “correct use and representation of time units” depends on two factors: (i) precise knowledge about time units and their relationships; (ii) the coupling of a time unit with changes that can occur during this time unit. The two scores proposed here seek to assess these two different dimensions of time knowledge. TKQ allows clinical implication to determine whether conventional time or estimated time could be affected in children with disorders or disabilities.

## Declarations

### Author contribution statement

F. Labrell: Conceived and designed the experiments; Performed the experiments; Analyzed and interpreted the data; Contributed reagents, materials, analysis tools or data; Wrote the paper.

G. Dellatolas: Conceived and designed the experiments; Analyzed and interpreted the data; Contributed reagents, materials, analysis tools or data.

H. Câmara Costa: Analyzed and interpreted the data; Contributed reagents, materials, analysis tools or data.

H. Perdry: Analyzed and interpreted the data.

### Funding statement

This research did not receive any specific grant from funding agencies in the public, commercial, or not-for-profit sectors.

### Competing interest statement

The authors declare no conflict of interest.

### Additional information

No additional information is available for this paper.

## Figures and Tables

**Table:** 

Score	Interview Duration (minutes)							
	10	15	20	25	30	35	40	45
0	Don't know 1mn >1hr40min	Don't know 30s to 1mn >2hr30mn	Don't know 30s to 2mn >3hr20mn	Don't know 30s to 2mn >5hr	Don't know 30s to 3mn >5hr	Don't know 30s to 3mn >6hr40mn	Don't know 30s to 3mn >6hr40mn	Don't know 30s to 4mn >8hr20mn
1	1hr20m to 1hr30mn	2hr–2hr20mn	1hr40mn to 2hr30mn	3mn 2hr20mn to 4hr10mn	4hr–4hr10mn	4mn 5hr	4mn to 5mn	5mn 6hr40mn
2	1hr10mn	2mn 1h40mn to 1hr50mn	3mn 2hr10mn to 2hr30mn	2hr40mn to 3hr	4mn 3hr20mn to 3hr50mn	5mn 3h50mn to 4hr10mn	6mn 5hr	6mn to 7mn 5hr
3	51mn to 1hr	1hr20mn to 1hr30mn	1hr50mn to 2hr	4mn 2hr10mn to 2hr30mn	5mn 2hr40mn to 3hr	6mn 3hr–3hr40mn	7mn 3hr30mn to 4hr10mn	8mn 3hr50mn to 4hr10mn
4	2mn 40mn to 50mn	3mn 1hr–1hr10mn	4mn to 5mn 1hr20mn to 1hr40mn	5mn to 6mn 1hr40mn to 2hr	6mn to 7mn 2hr–2hr30mn	7mn to 8mn 1hr20mn to 2hr50mn	8mn to 9mn 2hr40mn to 3hr20mn	9mn to 11mn 3hr–3hr40mn
5	3mn 32mn to 39mn	4mn 48mn to 59mn	6mn 1hr10mn	7mn 1hr20mn to 1hr30mn	8mn to 9mn 1hr40mn to 1hr50mn	9mn to 11mn 2hr–2hr10mn	11mn to 12mn 2hr10mn to 2hr30mn	12mn to 14mn 2hr30 to 2hr50mn
6	26mn to 31mn	5mn 38mn to 47mn	7mn 51mn to 1hr	8mn to 9mn 1h10mn	10mn to 11mn 1hr20mn to 1hr30mn	12mn to 13mn 1hr30mn to 1hr50mn	13mn to 15mn 1hr50mn to 2hr	15mn to 17mn 2hr–2hr20mn
7	4mn to 5mn 20mn to 25mn	6mn to 7mn 30mn to 37mn	8mn to 10mn 40mn to 50mn	10mn to 12mn 50mn to 1hr	12mn to 15mn 1hr–1hr10mn	14mn to 17mn 1hr10mn to 1hr20mn	16mn to 20mn 1hr20mn to 1hr40mn	18mn to 22mn 1hr30mn to 1hr50mn
8	6mn 16mn to 19mn	8mn to 9mn 24mn to 29mn	11mn to 12mn 32mn to 39mn	13mn to 15mn 40mn to 49mn	16mn to 18mn 48mn to 59mn	18mn to 22mn 56mn to 1hr	21mn to 25mn 1hr10mn	23mn to 28mn 1hr20mn
9	7mn 13mn to 14mn	10mn to 11mn 19mn to 23mn	13mn to 15mn 26mn to 31mn	16mn to 19mn 32mn to 39mn	19mn to 23mn 38mn to 47mn	23mn to 27mn 44mn to 55mn	26 to 31mn 51mn to 1hr	29mn to 35mn 57mn to 1hr10mn
10	8mn to 12mn	12mn to 18mn	16mn to 25mn	20mn to 31mn	24mn to 37mn	28mn to 43mn	32mn to 50mn	36mn to 56mn

## Appendix A Time Knowledge Questionnaire

### Subtests

***Orientation*** *(OR)*: (1) What day is it today? (2) What time is it*? (3) What month is it? (4) What year is it? (5) What season is it? [Maximum score = 5]

***Sequences*** *(SEQ)*: (1) What month comes after March? (2) Can you tell me the months in the year in order? (3) Can you tell me the seasons in the year in order? [Maximum score = 3]

***Time units*** *(TU)*: (1) Is a minute shorter or longer than a second? (2) Is a week longer or shorter than a month? (3) Is a month shorter or longer than a year? (4) How many hours are there in one day, from one day in the morning to the next day in the morning? [Maximum score = 4]

***Telling the time on a clock*** *(CL)* (six images of a clock): (1) Can you show me 2 o’ clock?; (2) Can you show me 10 to 3?; (3) Can you show me a quarter past 8?; (4) Can you show me 10 past 1?; (5) Look at this clock. How many minutes are there to 2 o'clock? [Maximum score = 5]

***Life span*** *(LS)* (four pictures: baby, child, young man, and old man): How long does it take: (1) for a baby (here) to become a child (here)? (2) for a child (here) to become a young man (here)? (3) for a young man to become an old man? [Maximum score = 6]

***Birthdays*** *(BIR)*: (1) ** How old are you? (2) ** When is your birthday? (3) How long ago was your birthday? (4) How long is it to your next birthday? [Maximum score = 6]

***Interview duration*** *(ID)*: (1) For how long have we been here together? [Maximum score = 10]

*Considered as correct if the child's answer is in the same half day (if it is 10 o'clock and the answer is "it is 8 o'clock” or "it is midday,” the child's answer is considered correct, but if the child's answer is "it is 4 o'clock in the afternoon”, it is considered incorrect).

** Questions not considered in the calculation of sub-scores and total score.

### Summary scores

***Summary score 1***: Sum of *OR*, *SEQ*, *TU* and *CL* [Maximum score = 17]

***Summary score 2***: Sum of *LS*, *BIR* and *ID* [Maximum score = 22]

***Total score***: Sum of all subtests [Maximum score = 39]

## Footnotes

1 See *La Pensée*, 1980, n°210, p.22. Report from the French Society of Philosophy in 1922 where A.Einstein answered a question from H. Bergson about links between psychology and physics.
